# Book Reviews

**Published:** 1873-01

**Authors:** 


### BIBLIOGRAPHICAL.

*The Pathology of the Teeth, with special reference to their Anatomy and Physiology.*—By Carl Wedl, M. D., Professor of Histology in the University of Vienna.—Translated from the German by W. E. Boardman, M. D.; with notes by Professor T. B. Hitchcock, M. D., D. D. S., of Harvard University. Published by Lindsay & Blakiston, Philadelphia.

Prof. Wedl in preparing this work on the pathology of the teeth, has supplied a want long felt by those interested in this subject, and shown himself to be a thorough and patient investigator; his deductions being drawn from personal observations and research. Pathology is a favorite study with the author of this work, as in 1855 the Sydenham Society published his "Rudiments of Pathological Histology;" a treatise which established the author's reputation as a distinguished scholar.

To Dr. W. E. Boardman, and Professor T. B. Hitchcock, are due much credit; the first for his literal translation of this work on "Pathology of the Teeth," from the German, and the second for the explanatory notes throughout its text, which add greatly to its value as a text-book to the dental student. The work is profusely illustrated with wood cuts, and gotten up in a style, which is common to the publications of the widely known publishers by whom it is issued.

Part I, consisting of some 97 pages, is devoted to the Anatomy and Physiology of the Teeth, and Growth of the Jaws.

Part II is devoted to Pathology; including 1, Irregularities in the Formation of the Teeth; 2. Inflammation of Pulp, Gums, Periosteum, etc.; 3. Atrophies; 4. Hypertrophies; 5. New Formations; 6. Anomalies of the Secretions; 7. Neuroses; ending with an alphabetical index.

Some two years since, the German edition of this work was published, succeeded by the "Atlas of the Pathology of the Teeth," with German and English explanatory text, by Prof. Heider and Wedl; which contains many valuable lithographs, to which reference is frequently made in the work we have under consideration.

Prof. Wedl in this work, attaches great importance to the influence possessed by the teeth over affections of the nervous system, respiratory organs, and organs of digestion, etc.

The interesting case of a fractured tooth uniting, is mentioned as coming under his own personal observation; in which the reparation process began in the pulp and dentine, in the form of globular masses. Prof. Wedl regards caries of the teeth as the result of abnormal secretions, and remarks, "In consequence of the decomposition of the secretions, acids are formed which extract the calcareous salts from the hard tissues, and give rise to a disintegration of the affected portions of the latter, in which no inflammatory reaction occurs. The destructive process is promoted essentially by the accumulations of secretions and particles of food, and opportunity is afforded for the proliferation of leptothrix buccalis in the dead and softened dentine."

As regards the shedding of the temporary teeth, Prof. Wedl thinks that the appearances indicate an interstitial absorption, the formation of openings in the cementum, which become filled with amorphous calcareous salts. The tissue most active in the destruction of the cementum, he describes as a granulation tissue, often containing a varying number of giant-cells, and probably developed from the root membrane of the temporary tooth, and from the medulla of adjacent osseous tissue. Where this is in contact with the root, depressions are produced similar to those seen upon necrosed bone. His opinion is that the cells of the granulation tissue secrete a fluid which dissolves the hard substance.

He regards salivary calculus as an organic cement of mucous epithelium. Mixed saliva, and leptothrix, united with the inorganic salts precipitated from the saliva, which thus forms the incrustation.

There are a great many other points of interest, which might be referred to if space permitted, which a perusal of the work will furnish to all interested, and which are invaluable to the dental student and practitioner.

---

*The Physician's Visiting List for* 1873.—Twenty-second edition. Lindsay & Blakiston, Philadelphia. While this is one of the most convenient lists for the physician, it is no less so for the dental practitioner as an engagement book; the manner in which it is arranged, answering such a purpose admirably. For a number of years we have used it in dental practice to record daily engagements, while the information it contains renders it valuable in cases of emergency, such as may arise from effects of poisons.

---

*Resections of the Maxillary Bones, without External Incision.*—Illustrated by cases, and a description of Instruments. By D. H. Goodwille, M. D., D. D. S. A pamphlet of 19 pages, reprinted from *N. Y. Medical Journal*, July, 1872.

*Lecture on Water.*—Delivered before the American Institute of the city of New York. By C. F. Chandler, Ph. D. A pamphlet of 48 pages containing valuable information, and illustrated with wood cuts.

---

*Ueber das Zahnen der Kinder.*—Von Adolph Petermann, D. D. S., Frankfort, 1872. A dissertation upon the origin and development of the temporary teeth, in German text and well written, in the form of a pamphlet of 14 pages.

---

*The American Agriculturist.*—This useful and attractive journal continues to hold the high position it has so justly merited among the leading agricultural publications of our time; having few equals and no superior. Every number reaches us with a profusion of finely executed wood engravings, illustrating the text, and rendering it more attractive and interesting. This journal also contains matter apart from the farm and household, that causes it to be a welcome monthly visitor to the young as well as to the old. Snbscription price, $1.50 a year. Published by Orange, Judd & Co., 245 Broadway, N. Y.

---

*Wood's Household Magazine.*—The January number, 1873, of this interesting and instructive monthly, has been received, and contains good original and selected matter from foreign books and periodicals. This publication has recently been remodeled, and considerably improved in appearance, and has now reached its twelfth volume.

The January number contains the following articles: One Cause of Trouble, by Gail Hamilton; Catherine's Christmasses, by Harriet Prescott Spofford; Co-operative Stores, by Sidney Hyde; Good Cheer, by E. D. Rice; General William Wirt Colby, by Rebecca Harding Davis; Reading aloud; Song of the Sordid Sweetheart; The Rooster-Pecked Wife; The Law of Courtship; The House of Mourning; Agostina, the Maid of Saragossa; Story of the Sand Man; Peace on Earth; Peep: A Fragment for the Young; Forfeits; The Spider and the Flea; Editorials, including: Remodeling, Holiday Greetings, Literary Review, Fashions, Housekeeper, Sense and Nonsense, and Love Thoughts of eminent persons. Subscription price, one dollar a year. S. S. Wood, Newburgh, N. Y.

